# Does intra-articular fracture change the lubricant content of synovial fluid?

**DOI:** 10.1186/s13018-015-0232-6

**Published:** 2015-06-03

**Authors:** Hasan H. Ceylan, Mehmet Erdil, Gokhan Polat, Deniz Kara, Elif Kilic, Abdurrahim Kocyigit, Ibrahim Tuncay

**Affiliations:** Department of Orthopaedics and Traumatology, LNB State Hospital, Istanbul, Turkey; Department of Orthopaedics and Traumatology, Istanbul Medipol University Medical Faculty, Istanbul, Turkey; Department of Orthopaedics and Traumatology, Istanbul University Medical Faculty, Istanbul, Turkey; Department of Orthopaedics and Traumatology, Bezmialem Vakif University Medical Faculty, Istanbul, Turkey; Department of Clinical Biochemistry, Bezmialem Vakif University Medical Faculty, Istanbul, Turkey

**Keywords:** Hyaluronan, Proteoglycan, Lubricant, Synovial fluid, Fracture

## Abstract

**Background:**

Lubrication function is impaired and the lubricant content of synovial fluid (SF) changes immediately after plateau tibia fractures. Here, we aimed to analyze the lubricant content of SF at chronic term following plateau tibia fracture.

**Methods:**

Forty-eight surgically treated patients without joint incongruency (<2 mm displacement) were included in the study. Joint aspiration had been possible in 16 of the participants. However, sampling could be made from healthy knees in only ten of these patients. Twenty-six SF samples (16 injured knees, 10 healthy knees) were analyzed for concentrations of hyaluronic acid (HA), proteoglycan-4 (PRG4), TNF-α, IL-1β, and IL-6.

**Results:**

The group of experimental samples were obtained at a mean of 31 (12–66) months after injury from patients with a mean age of 45.1 (32–57) years. There were no relationships detected between biochemical analysis results and patient ages, sexes, postoperative time, and fracture type. After excluding six patients for whom we could not sample from their healthy knee, ten patients’ values were compared with paired Wilcoxon signed rank test and no significant differences detected between the healthy and injured knee in terms of the SF concentrations of HA and PRG4 (*p* = 0.225 and 0.893, respectively). Similarly, there were no statistically significant differences in SF sample concentrations of TNF-α, IL-1β, and IL-6 between healthy and injured knees.

**Conclusions:**

Despite acute changes, the long-term concentrations of HA and PRG4 were similar after plateau tibial fracture. We could not detect any concentration level differences between healthy knees and injured knees regarding HA and PRG4 in the long-term follow-up.

## Background

Intra-articular fractures are disabling injuries that deteriorate the articular surface [1, 2]. Deterioration and insufficient restoration of articular surfaces may cause arthrosis and secondary labor loss [2].

Apart from the articular surface integrity, an ideal lubrication provides reduction of friction and wear of the cartilage surface. This mechanism is directly related to lubricant content of synovial fluid (SF) with hyaluronic acid (HA) and proteoglycan-4 (PRG4) [3]. Lubrication activity of SF is critical because even small increases in friction may result in disruption of joint surfaces [4]. HA and PRG4 are the primary lubricant macromolecules in SF [5], with HA being the quantitatively major component. HA is characterized by its high bulk viscosity and is a known lubricant agent capable of reducing joint friction.

After intra-articular traumas, such as anterior cruciate ligament (ACL) injury and plateau tibia fractures, it is known that lubrication function is impaired, for example, PRG4 lubricant content decreases and degenerative enzymes and inflammatory marker levels increase [6]. In this way, cartilage destruction develops, and arthrosis starts. Similarly, after acute ACL injuries, the amount of lubricant is decreased in various animal models [7–9].

Here, we aimed to analyze the lubricant content of SF of patients who had a minimum of 12 months follow-up after being surgically treated for plateau tibia fracture.

## Methods

After local ethics committee approval (Bezmialem Vakif University Ethical Committee of Clinical Trials, approval no: B.30.2.BAV.0.05.05/518), the retrospective records of 126 tibia plateau fracture patients were analyzed. The patients, who had closed fractures and that had been surgically treated with a minimum follow-up of 12 months, were included in our study. All of the subjects were informed about our study and provided informed consent. All steps of the study were carried out in compliance with the Helsinki Declaration.

The data were analyzed using SPSS 11.5 (Statistical Package for Social Sciences) software. The student’s *t* test was utilized to examine statistical differences between injured and non-injured knee samples with significance reported at the 95 % confidence levels.

### Patient selection

Of the 126 patients, only 69 matched the inclusion criteria, with minimum follow-up of 12 months and surgically treated closed plateau tibia fracture. Twenty-one patients did not accept to participate in the study.

First, patients were examined by two clinicians to ensure that there was no acute injury condition. This study was primarily limited to those patients who had no history of receiving intra-articular injections prior to study and no recent joint traumas within 6 months. Seventeen patients were excluded because they matched one or more of the following: previous trauma to the non-injured knee; joint incongruity >2 mm in their postoperative computerized tomography (CT) scans; oral chondroprotective agent usage; history of previous knee infection; history of previous knee arthroscopy; with known viral diseases (HIV, Hepatitis B, and C) which may effect the serological results; and patients unable to provide consent. The remaining 31 patients composed the study group.

### Radiological examination

In accordance with ethics committee consent, CT scans were performed and the step-in joint surface was noted. Tibial articular surface displacement was measured digitally on CT images by two orthopedic surgeons by a digital imaging system (Centricity PACS, GE Healthcare, Pittsburg, PA). Fracture types were recorded according to the initial plain radiographs. Patients with joint incongruity >2 mm were excluded from the study.

### Synovial fluid sampling

The remaining 31 patients were scheduled for synovial sampling. All of our patients were sampled from both their injured and healthy knee. After preparation of a sterile sampling setting, a standard 20-gauge needle attached to a 10-cc syringe barrel was introduced into the joint and a minimum of 2 cc SFs was aspirated. Patients that could not be sampled from their injured knee were excluded from the study. Joint aspirations were successful in 16 patients, and these patients make up the study group. However, sampling could be performed from the healthy knee in only 10 of these 16 patients.

### Gross and biochemical analysis of human synovial fluid samples

**Fig. 1:**
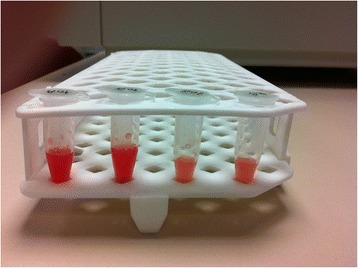
Synovial fluid. Gross appearance of collected human synovial fluid from both knees of a patient

**Fig. 2:**
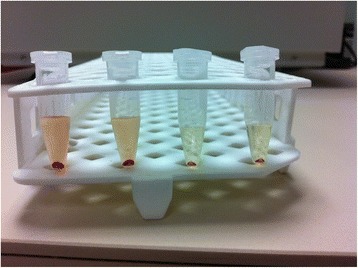
Before analysis. After centrifugation, the supernatant fluid including synovial content, and the bottom layer is the cell pellet of the sample

Any specific color change or opacity was not preferred for quality measurements. Therefore, SF samples were centrifuged (3000 *g* for 30 min at 4 °C) to obtain separate fractions (Figs. 1 and 2). Following centrifugation, the supernatants were separated and aliquots stored at −80 °C until analysis.

Twenty-six SF samples were analyzed biochemically for concentrations of HA, PRG4, tumor necrosis factor-α (TNF-α), interleukin-1β (IL-1β), and interleukin-1 (IL-6). A minimum number of two replicate samples per specimen were used for concentration measurements. The concentration of HA, TNF-α, IL-1β, and IL-6 in SF samples were determined by an enzyme-linked immunosorbent assay (ELISA)-like assay, and the PRG4 levels were determined by Western blot technique.

### Hyaluronan analysis

The concentrations of HA molecules in SF were determined by an ELISA-like assay, according to protocols provided by manufacturers (Hyaluronan ELISA kit, RD systems, Minneapolis, MN). The results were expressed in milligram/milliliter.

### PRG4 analysis

The concentration of PRG4 in SF samples was quantified by Western blot with the use of an antibody to lubricin after SDS-horizontal agarose gel electrophoresis on 3-mm-thick agarose (2 %) gels and transfer to polyvinylidene fluoride membrane. The immunoreactive proteins were detected by Amersham ECL Plus detection and digital scanning with a STORM 840 imaging system (GE Healthcare, Piscataway, New Jersey). ImageQuant (GE Healthcare) was used to generate densitometric scans. Like the previous studies, PRG4 was quantified with the use of purified explants and run on the same gels [10, 11]. The results were expressed in microgram/milliliter.

### TNF-α, IL-1β, and human IL-6 analysis

The concentrations of human TNF-α, IL-1β, and human IL-6 in SF were calculated using ELISA kits, according to protocols provided by manufacturers (Human TNF-α Platinum, Human IL-1β Platinum, and Human IL-6 ELISA kits, MedSystems, Vienna, Austria). The results were expressed in pg/mL.

## Results

A total of 26 fluid samples collected from 16 patients were used in this study. No other samples of control SF were used outside of the study group. Four of the patients were male and 12 were female. Mean age was 45.1 (32–57) years, and the mean follow-up period was 31 (12–66) months.

In preoperative examinations of digital recordings, three patients were found to have had: type 2, four patients; type 3, two patients; type 4, three patients; type 5, four patients; and type 6 tibia plateau fracture according to the Schatzker classification. In all patients, surgical treatment was acceptable (under 2-mm step-off) with restoration of the articular surface. The injured joint surfaces were collapsed by a mean of 0.39 mm (min. 0.13, max. 0.65, SD: 0.19), as detected by CT analysis on the day of SF sampling. In the analysis of Spearman’s non-parametric test, there was no significant correlation between the results of the biochemical analysis, fracture type, and displacement of articular surface (*p* > 0.05).

In biochemical analyzes of HA, TNF-α, interleukin-1B, interleukin-6, and PRG4 levels were compared with the amount of displacement of fractures that were evaluated with CT analyses, age, sex, and postoperative time. Using the Spearman’s correlation test, we detected no relationships between biochemical analysis results and patient ages, sexes, postoperative time, or Schatzker type parameters. There were no statistically significant differences between these parameters (*p* > 0.05).

After excluding six patients for whom we were unable to sample from their healthy knee, ten patients’ values were compared using the paired Wilcoxon signed rank test. There were no significant differences between the healthy and injured knee in terms of the composition of SF (*p* > 0.05). In this group, the average HA concentrations for the injured knee was 0.843 mg/mL (min. 0.78, max. 0.9, SD = 0.458) compared to 0.901 mg/mL for the injured knee (min. 0.829, max. 0.961, SD = 0.48, *p* = 0.225). The injured knee PRG4 average was 58.94 μg/mL (min. 50.7, max. 70, SD = 7.12), while the healthy knee PRG4 average was 58.82 μg/mL (min. 52.2, max. 69, SD = 6.35, *p* = 0893). Similar to above, there were no statistically significant differences in SF sample concentrations of TNF-α (*p* = 0.686), IL-1β (*p* = 0.225), and IL-6 (*p* = 0.225) between healthy and injured knees (Table 1).

**Table 1:** Analysis of inflammatory cytokines

	TNFalpha-h (pg/ml)	TNFalpha-i (pg/ml)	IL1B-h (pg/ml)	IL1B-i (pg/ml)	IL6-h (pg/ml)	IL6-i (pg/ml)
Min.	54.56884484	51.59461292	9.396551724	9.123152709	18.79310345	18.24630542
Max.	59.08147257	59.95323021	9.943349754	19.408867	19.88669951	38.81773399
Mean all	56.48671162	56.37004994	9.722660099	11.94488916	19.4453202	23.88977833
SD all	1.882955702	2.537548259	0.208957888	4.501883044	0.417915774	9.003766086
Mean-paired	56.48671162	56.55850343	9.722660099	9.594581281	19.4453202	19.18916256
SD-paired	1.882955702	3.061669654	0.208957888	0.091707019	0.417915774	0.183414039

The minimum (Min.), maximum (Max.), and mean and standard deviation (SD) values of inflammatory cytokines in synovial fluid samples (h, healthy and i, injured knees)

After the comparison of SF analysis results, no statistically significant differences were observed in SF sample concentrations of HA, PRG4, TNF-α, IL-1β, and IL-6 between healthy and injured knees. The mean HA level of all injured knees was identified as 0.841 mg/mL (min. 0.78, max 0.9, SD = 0.354). When these results were compared with the mean level of HA that was obtained from healthy knees, there was no significant difference (*p* = 0.225). Similarly, there was no significant difference between the mean level of PRG4 (59.51 μg/mL, min. 50.7, max. 70, SD = 5.84) taken from all injured knees and the mean level of PRG4 in healthy knees. In addition, there was no significant difference in inflammatory cytokine levels between healthy and injured knees (*p* > 0.05).

## Discussion

Tibial plateau fractures are intra-articular fractures that may lead to arthritis due to disruption of the articular cartilage through primary impact or inaccurate reduction of fracture. In addition, lubrication in the joint may be disturbed due to the change of SF characteristics. In this study, we attempted to analyze the concentrations of HA and PRG4 in patients who had been treated due to tibial plateau fractures and had higher risk for arthrosis in the long term.

Here, we report HA concentrations in the SF of healthy donors of between 1.8 and 3.33 mg/mL [11–14]. This compares with the slightly higher previously reported SF HA concentrations between 3.2 and 4.1 mg/mL [5]. There is as yet no consensus regarding the change in HA levels of SF under pathologic conditions, such as in osteoarthritis (OA) and intra-articular fractures, although some authors have reported normal levels of HA in arthritic joints [13, 15]. Ludwig et al*.* measured the SF HA concentrations of normal and osteoarthritic knees as 0.11 to 0.96 and 0.23 to 2.69 mg/mL, respectively [12]. The SF HA concentration in OA patients was reported as between 1.2 and 2.2 mg/mL [5]. According to one study, intra-articular fractures cause a decrease in HA levels in SF [11, 16]. Ballard et al*.* reported the mean HA levels of injured knees as 0.27 mg/mL [11]. Here, we detected the HA concentration of SF HA in healthy and injured knees as 0.841 and 0.901 mg/mL, respectively (*p* = 0.225), which are comparable with those reported in the literature. Although some parameters (e.g*.*, age, sex, trauma severity, and assay sensitivity) may affect these analyses [11], the concentration of HA in these samples did not differ between healthy and injured knees.

A deficiency in PRG4 proteins (also known as lubricin, superficial zone protein, and megakaryocyte-stimulating factor [10, 17]) is an important issue because it is known to increase friction at the joint surface [4, 11]. The reported PRG4 concentration in the SF of humans varies between 52 and 350 μg/mL postmortem and 276 to 762 μg/mL in SF obtained during arthrocentesis [11]. Ludwig et al*.* detected the PRG4 levels in normal SF as being between the ranges of 129 and 450 μg/mL [12]. There is a large variability in the measured PRG4 levels of SF from patients with joint disease [11, 12]. Changes in PRG4 concentration after acute injury have been previously noted [6, 11, 12, 18];, however, conflicting result regarding this have also been produced [6, 18]. In an animal study, the PRG4 concentration of SF decreased from 280 to a 20 to 100 μg/mL range at 3 weeks after injury [19]. These decreased PRG4 levels returned to normal values within 1 year of ACL injury [6]. Yet other studies have reported increased PRG4 concentrations after intra-articular fracture and OA patients [11, 18]. Additionally, in another study, increased PRG4 concentrations were found to be correlated with the severity of OA [18]. These differences may be related to the study design, biochemical assay, and objective selection criteria [11]. In our study, all of the samples were taken from the same patient population (i.e*.*, both injured and healthy joints were exemplified with same biochemical assay). The mean PRG4 concentrations were 59.51 μg/mL in injured knees and 58.82 μg/mL (*p* = 0.893) in healthy knees, according to our analyses. This similarity in the mean levels of the injured and healthy knees espoused the hypothesis that PRG4 levels return to normal in the long-term analyses [6].

A previous animal study demonstrated that increased intra-articular fracture severity increases acute joint pathology, including synovial inflammation [20]. Furthermore, pro-inflammatory cytokines such as IL-1, IL-6, and TNF-α were upregulated in injured and degenerative joints [21, 22]. SF levels of IL-1β and TNF-α were measured by ELISA to exclude the effects of the acute injury, which may affect the results. The presence of inflammatory cytokines (IL-1β, TNF-α) in the SF samples was quantified by commercially available kits. To exclude the effects of new trauma and inflammation, which may affect the PRG4 levels, we controlled the IL-1β and IL-6 levels [6].

SF is a combination of HA, PRG4, and surface active phospholipids (SAPL); each of these molecules interact for boundary lubrication function of SF [23]. Although HA and PRG4 are the primary lubricant macromolecules, conflicting results regarding their normal concentrations have been reported [5]. We identified no difference in the HA and PRG4 concentrations of healthy and injured knees. Although we could not make a mechanical test for friction change, we think that lubrication change may be due to another factors like unknown molecules other than HA or PRG4.

Ballard et al*.* reported that knees afflicted with a tibia plateau fracture have SF with decreased lubrication properties, as well as decreased concentrations of HA [11]. In Ballard et al.’s study, the samples were taken during the fracture operation and no long-term or time dependent changes were taken into consideration [11]. Instead, they showed the correlation between lower HA and lower lubrication properties of pathological SF; however, this condition appears to recover after 1 year. These findings are in line with the results reported here.

Ross et al*.* reported a comparative study on injured and uninjured knees [24], which included 52 patients, but the study group was not homogenous and included both acute and previous trauma cases. Aspirations from injured and non-injured knees were equally successful in all cases. They used ten healthy athletes from among the injured sampling group as a reference group. We propose that this might have led to a bias in their study. In our study, all of the samples were taken from the same patient population (i.e*.*, both injured and healthy joints were exemplified with same biochemical assay).

It has been reported that, in the case of acute trauma, increased loss through the inflamed synovium may account for the low levels of PRG4 [5, 25]. However, in the study of Ballard et al. the results were controversial, with the HA levels also being lower. This decrease in HA concentration may be due to SF dilution caused by traumatic edema. Further studies are needed to elucidate these issues.

The results of our study indicated that the chronic term concentrations of HA and PRG4 in SF are similar after plateau tibial fracture. Various studies have reported results contrary to this finding [5, 6, 11, 12, 18, 19]. We used the uninjured knees of patients to check for the presence of active inflammation by IL-1β, IL-6, and TNF-α and confirmed the absence of current trauma, which may have affect the results. We could not find any concentration level differences between healthy knees and injured knees regarding HA and PRG4 within a minimum of 1 year follow-up. Furthermore, the mediators (IL-1β and IL-6) related to arthrosis were not quantitatively higher in fractured knees.

Our study has some limitations. First, the sample size was limited due to some patients not consenting to participate to our study and other restrictions due to inclusion criteria. We were therefore unable to make a subgroup evaluation of this patient group due to limited sample size. We would also obtain more objective results if we could make the same analysis for these patients in the acute and chronic term. On the other hand, a power analysis was not performed before the study. A prospective study, with a prior power analysis may give more relevant information in this topic. Also, sampling of the highly viscous SF is difficult; in six patients, we were unable to aspirate SF from their healthy knees. Additionally, there might be increased stress on the contralateral knee in patients with injury that could alter the synovial fluid and the HA and PRG4 results may be affected. Another limitation is that we were unable to make a molecular weight analysis of HA samples. In addition, we were unable to make a lubrication analysis due to financial and technical impairments. Synovial proteins (e.g*.*, COMP and lubricin) that may affect lubrication were not analyzed [10, 17]. Systemic levels of HA and inflammatory cytokines, that have been shown to predict disease outcome, were also not analyzed [26, 27]. We sampled the healthy knee of each patient. But the unaffected knee may be overly stressed due to the fact that the patient had an injury on the contralateral side and, therefore, would not be completely normal due to the increased stress from compensating for problems on the contralateral side. The concentrations of each protein may change at various moments after an injury; repetitive analyses may help detailed analysis of this problem. More detailed studies are required to better demonstrate the accurate changes in SF composition.

## Conclusions

Knee homeostasis seems to be restored in terms of the synovial fluid 1 year following a traumatic event. Further prospective studies that combine the biochemical and biomechanical analyses of SF may be helpful in clarifying these issues in the chronic posttraumatic period.
